# Modified Vitrectomy Technique for Phakic Rhegmatogenous Retinal Detachment with Intermediate Break

**DOI:** 10.1155/2018/6127932

**Published:** 2018-10-23

**Authors:** Vincenza Bonfiglio, Mario D. Toro, Antonio Longo, Teresio Avitabile, Robert Rejdak, Katarzyna Nowomiejska, Tomasz Choragiewicz, Andrea Russo, Matteo Fallico, Agnieszka Kaminska, Elina Ortisi, Stefano Zenoni, Michele Reibaldi

**Affiliations:** ^1^Eye Clinic, University of Catania, Catania, Italy; ^2^Department of General Ophthalmology, Medical University of Lublin, Lublin, Poland; ^3^Institute for Ophthalmic Research, University Eye Hospital, Tuebingen, Germany; ^4^Faculty of Family Studies, Cardinal Stefan Wyszynski University, Warsaw, Poland; ^5^Life Clinic, Milano, Italy

## Abstract

**Purpose:**

To evaluate the effects of a modification of the traditional 25-gauge pars plana vitrectomy technique in the treatment of uncomplicated macula-on rhegmatogenous retinal detachment (RRD) with intermediate retinal break(s) and marked vitreous traction in the phakic eye.

**Methods:**

Prospective, noncomparative, and interventional case series. All consecutive phakic eyes with primary uncomplicated macula-on RRD with intermediate retinal break(s) and marked vitreous traction, with at least 1 year of postoperative follow-up, were enrolled. In all eyes, “localized 25-gauge vitrectomy” under air infusion with localized removal of the vitreous surrounding the retinal break(s), in association with laser photocoagulation and air tamponade, was performed. The primary end point was the rate of primary retinal attachment. Secondary end points were cataract progression and assessed by digital Scheimpflug lens photography (mean change of nuclear density units) and the rate of complications.

**Results:**

Thirty-two phakic eyes were included in the final analysis. At 12 months, the primary outcome of anatomical success was achieved in 94% of eyes. The mean nuclear density units did not change significantly at any time point during the follow-up. After localized vitrectomy, one eye developed an epiretinal membrane, and one eye developed cystoid macular edema; no other significant complications were reported.

**Conclusions:**

“Localized vitrectomy” has a high anatomical success rate in phakic eyes with primary uncomplicated macula-on RRD with intermediate retinal break(s) and marked vitreous traction, without causing progression of cataract.

## 1. Introduction

Scleral buckling (SB), primary pars plana vitrectomy (PPV), and pneumoretinopexy (PR) are the surgical procedures to treat primary rhegmatogenous retinal detachment (RRD). In the last few decades, primary PPV is the method of choice to manage RRD for several reasons including technical advances, lower postoperative inflammation, less patient discomfort, and greater familiarity of surgeons with this technique compared to the SB procedure [1–3]. The major disadvantages of primary PPV are cataract progression and iatrogenic retinal breaks [1].

The location and size of the retinal break(s) is one of the clinical features that influence the choice of treatment [1].

The scleral buckling versus primary vitrectomy in rhegmatogenous retinal detachment (SPR) study [4] included primary medium-severe RRD with intermediate breaks, described as “breaks between the equator and major vessel arcades.”

In the management of RRD, the SPR study [5] suggested that the SB procedure in the phakic eyes shows a better postoperative visual acuity while the vitrectomy technique in the pseudophakic eyes shows better anatomical outcomes. However, no correlation about choice of treatment between SB and PPV, and between visual and anatomic outcomes, according to the location of breaks was made in this study specifically.

SB is difficult to perform in cases involving an intermediate location of the break(s), and it is associated with many possible complications [6–10].

However, SB has the advantage of less risk of cataract development and substantially lower cost over PPV [1, 11].

In this study, the authors describe a new technique called “localized vitrectomy,” used to treat uncomplicated macula-on primary RRD with intermediate break(s) and marked vitreous traction, in the phakic eyes. This procedure is a modification of the traditional 25-gauge PPV, consisting of a mini-invasive vitrectomy with a limited vitreous removal surrounding the retinal break(s), without core vitrectomy or shaving the vitreous base over 360°. Furthermore, the authors have evaluated the efficacy of this procedure, including visual and anatomic results, complication rate, and postoperative cataract progression.

## 2. Methods

In this prospective study, all consecutive phakic eyes that underwent 25-gauge PPV for primary macula-on primary RRD with intermediate break(s) and marked vitreous traction at the Ophthalmological Clinic of Catania between January 2014 and September 2016 were included. The risks and benefits of the treatment were explained to the patients, and a written consent was obtained in accordance with the Helsinki Declaration before the procedures. The Institutional Review Board/Ethics Committee approved the design of the study.

The inclusion criteria were as follows:

1. Phakic eye
2. Primary uncomplicated macula-on RRD including PVR grade A or B with one or more contiguous intermediate retinal break(s), defined as breaks between the equator and major vessel arcades [2], and with marked vitreous traction
3. Presence of posterior vitreous detachment (PVD)
4. Absent-to-moderate cataract (grade 0.0 to 2.0 in the Thompson classification) in the RRD eye and in the controlateral eye [12]
5. Minimum follow-up of 12 months

Patients were excluded if they had secondary retinal detachment, previous ocular surgery, amblyopia, other rhegmatogenous retinal lesions, posterior retinal breaks (macular hole or between the major vessel arcades), giant breaks, or vitreous hemorrhage that required complete PPV.

Primary end point was the rate of primary retinal attachment; secondary end points were cataract progression and the rate of complications.

Two experienced vitreoretinal surgeons evaluated all primary RRD at the Ophthalmological Clinic of Catania between January 2014 and September 2016 and independently assessed and identified macula-on primary RRD with intermediate break(s) and marked vitreous traction. All cases in which surgeons differed in their clinical assessment of degree of the RRD, with inconsistence decisions, were excluded.

We divided fundus drawings into 4 quadrants centered at the fovea, superotemporal (ST), superonasal (SN), inferotemporal (IT), and inferonasal (IN), respectively, and recorded the location of each break in the 4 quadrants.

As superior break, we defined a retinal break located between 9 and 3o'clock meridian, and as inferior break, a retinal break between 4 and 8o'clock meridian.

Before vitrectomy, an independent, experienced retinal specialist (M. F.) assessed the presence of PVD using a slit-lamp biomicroscopy with an external lens of 78 diopters to identify the presence of the Weiss ring and the visible posterior vitreous cortex. A second retinal specialist (A. R.) performed 10 MHz B-scan ultrasonography (Cinescan S HF, Quantel Medical, Clermont-Ferrand, France) using transverse and longitudinal scans. Only eyes with PVD confirmed by both techniques were enrolled in the study.

All patients underwent a complete ophthalmic evaluation including measurement of best-corrected visual acuity (BCVA) and intraocular pressure (IOP) and examination of the anterior segment and dilated fundus preoperatively (at baseline) and at 1 day, 1 week, and 3, 6, 9, and 12 months after surgery.

BCVA was measured using early treatment diabetic retinopathy study charts by a single well-trained and experienced ophthalmologist (M. T.). Vision results were quantified as a logarithm of the minimum angle of resolution (logMAR).

IOP was measured by the Goldmann applanation tonometry. Hypotony was defined as an IOP of 5 mmHg or less.

In all patients, the lens status evaluation was performed with digital Scheimpflug lens photography at the baseline and at 3, 6, and 12 months after surgery. The nuclear density was assessed in the vitrectomized eye (study group) and in the fellow eye (control group). Lens images were obtained and analyzed by using a Nidek EAS-1000 anterior segment analysis system (Nidek, Gamagori, Japan). All lens images were taken by the same observer (A. L.) after pupillary dilation and at the same settings, as previously described by Sawa et al. [13] and Vivino et al. [14]. The opacification value of the nuclear region was expressed in nuclear density units (NDUs).

All preoperative, intraoperative, and postoperative data including patient demographics (age and sex) and postoperative complications were recorded in a database. The incidence, timing, and causes of retinal redetachment were also registered.

### 2.1. Surgical Technique

All patients underwent 3-port 25-gauge vitrectomy with a valved trocar system performed by the same surgeon (T. A.) under local sub-Tenon's anesthesia (using 10 ml of a 50 : 50 mixture of 2% lidocaine and 0.5% bupivacaine with 150 IU hyaluronidase). Surgical procedures were performed using the Stellaris PC under a Resight 700 noncontact panoramic viewing system (Carl Zeiss Meditec). The sclerotomy was placed 4 mm posterior to the limbus. With closed infusion, the retinal break(s) were localized, the eye was rotated in order to position the region of the retinal break as high as possible, and air infusion was started with a pressure of 30–35 mmHg. Localized removal of the vitreous surrounding the retinal break(s) was performed, and a complete release of the vitreoretinal adhesion surrounding the retinal break(s) was obtained. Finally, the subretinal fluid was drained with a needle through the retinal break. Neither core vitrectomy nor shaving of the vitreous base was performed.

After complete retinal attachment was achieved, endolaser photocoagulation was applied around the retinal break(s). Tamponade was performed with filtered air. Transconjunctival sutures were placed only in two eyes, in which leakage at the sclerotomy sites was observed. All patients were asked to maintain a specific head position, according to the location of the retinal break, for 3 days after surgery. In particular, patients with inferior break(s) were instructed to maintain a face-down and lateral position, while patients with superior break to maintain upright and lateral position depending on the quadrant o'clock meridian.

### 2.2. Statistical Analysis

Measured Snellen visual acuity values were converted to the logMAR values for subsequent analysis. The analysis of variance (ANOVA) was used to compare the mean values of pre- and postoperative BCVA and IOP in the vitrectomized eyes (study group) and to compare the mean NDUs of the study group eyes with that of the control group eyes (fellow eyes) at baseline and at 3, 6, and 12 months after treatment. Multiple comparisons were performed using the Tukey HSD test, if the differences were significant. Student's *t*-test was used to compare the mean NDUs detected in the two groups. *P* values <0.05 were considered significant. The data were analyzed using the Statistical Packages for the Social Sciences for Windows (v.17.0; SPSS, Chicago, IL, USA).

## 3. Results

**Figure 1:**
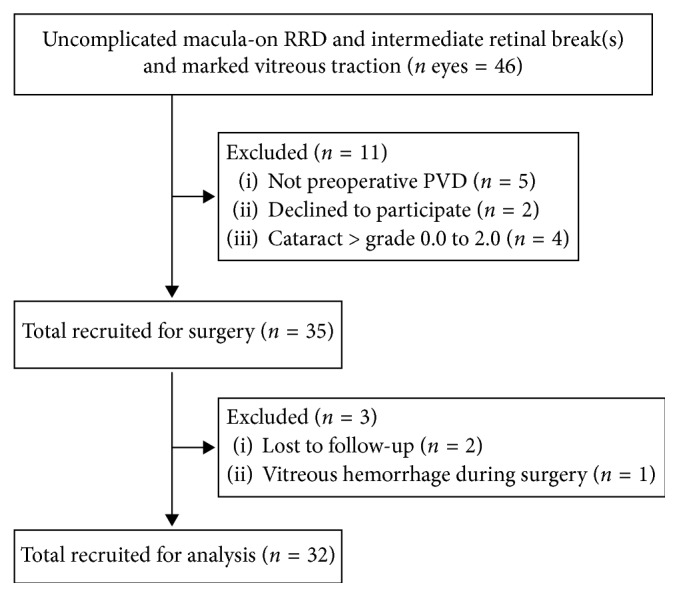
Flow diagram of the study (enrollment).

Of the 46 phakic consecutive eyes with uncomplicated macula-on RRD and intermediate retinal break(s) with marked vitreous traction, 11 eyes were excluded (5 eyes did not have PVD, 4 eyes had cataract more than grade 0.0–2.0, and 2 patients declined to participate), and 35 eyes addressed the inclusion criteria and were enrolled in the study. Of the these 35 eyes recruited for surgery, only 32 eyes were included in the analysis because 2 patients were lost during the follow-up period, and one patient had intraoperative vitreous hemorrhage during surgery and required conversion to standard PPV (Figure 1). Of the 32 eyes with RRD, 22 (68.7%) had superior retinal breaks and 10 (31.3%) had inferior retinal breaks. In particular, 19 eyes (59.3%) had retinal break(s) located in the ST quadrant, 7 eyes (21.8%) had break(s) in the IT quadrant, 3 eyes (9.3%) in the SN, and 3 eyes (9.3%) in the IN quadrant.

The mean (SD) age of patients was 61.5 ± 13.3 years; 18 patients (56%) were men, and 14 (44%) were women.

### 3.1. Primary Anatomical Success Rate

The primary anatomical success rate, defined as retinal reattachment at the final follow-up of 12 months after a single operation, was 94% (30 of 32 eyes): 95 4% of eyes with superior retinal breaks (21 of 22 eyes) and 90% of eyes with inferior retinal breaks (9 eyes of 10), respectively (*P*=0.534). Recurrence of RRD occurred in 2 eyes (6%) during the follow-up period: one eyes with preoperative ST retinal break and one eye with preoperative IT retina break. Both occurred within 1 month after the first operation. The redetachment was attributed to development of grade C PVR in one eye and to a new peripheral retinal break in the inferior quadrant in the other eye. Both of these eyes were reoperated by 25-gauge vitrectomy and SF6 tamponade; the retinal reattachment was obtained in both eyes. Thus, the final anatomical success rate, defined as retinal attachment at the final follow-up without regard to additional procedures, was 100% (32 of 32 eyes).

### 3.2. Visual Acuity

ANOVA showed no change in the mean BCVA from the baseline to 12 months after surgery. The mean ± SD logMAR BCVA was 0.17 ± 0.13 logMAR, 0.17 ± 0.12 logMAR, 0.16 ± 0.12 logMAR, and 0.16 ± 0.1 logMAR, respectively, at the baseline and 3, 6, and 12 months, without significant difference (ANOVA, *P*=0.973).

### 3.3. Progression of Lens Opacity

At the baseline, the mean ± SD NDU was 68 ± 12 in the study group and 69 ± 14 in the control group (*t*-test *P*=0.933) (Table 1). ANOVA showed that the mean NDUs did not change significantly in either group during the follow-up (study group *P*=0.523; control group *P*=0.725). No difference in NDUs was found between two groups at 3, 6, and 12 months.

No intraoperative complications were observed.

No significant IOP changes were detected during the follow-up period (ANOVA, *P*=0.781). The mean ± SD preoperative and postoperative IOP at 1 day, 1 week, and 1, 3, 6, and 12 months, were 13.6 ± 3.1 mmHg, 13.1 ± 4.5 mmHg, 13.7 ± 3.2 mmHg, 14.2 ± 5.2 mmHg, 14.7 ± 4.7 mmHg, 14.4 ± 3.9 mmHg, and 14.2 ± 4.5 mmHg, respectively. No hypotony was detected in any eyes, and none of the patients had endophthalmitis after surgery.

One eye developed an epiretinal membrane (ERM) 3 months after surgery and one eye showed cystoid macular edema at 1 month-follow up examination, resolved after topical therapy. No other postoperative complications were registered.

## 4. Discussion

Our study showed that a “localized vitrectomy” was effective in the treatment of primary macula-on RRDs, with superior and inferior, intermediate break(s) and marked vitreous traction in the phakic eyes and did not cause significant progression of cataract.

To date, no study has evaluated the efficacy of different surgical techniques in presence of uncomplicated RRD and intermediate retinal break(s).

PR is a well-accepted alternative surgical technique to scleral buckling and vitrectomy for RRDs with one or more retinal breaks within one clock hour; however, it is contraindicated in the eyes with inferior retinal break(s) and in which breaks are held open by vitreous traction [15].

The SPR study evaluated the phakic eyes with medium-severe primary RRD and intermediate break(s) [3] and shows better functional outcomes with the SB procedure than PPV. However, SPR study analysis included primary RRD with many different preoperative variables, such as macula-on only in 42.9% of eyes, RD with multiple breaks in different quadrants, bullous RD, intermediate breaks with marked vitreous traction, and RD with unclear hole situations. Furthermore, in the SPR study, no subanalysis of postoperative outcomes was conducted to identify any relation between choice of treatment and location of break(s), in particular intermediate break(s) [3].

The surgical SB procedure to treat primary RRD is very challenging in the eyes with intermediate break(s). Despite the advantages of not increasing the risk of cataract and being less expensive than PPV, SB can cause several possible complications, such as myopic shift in refraction (68%) [6], diplopia with extraocular muscle dysfunction (3%–50%) [6–8], choroidal detachment (23%–44%) [7], subretinal hemorrhage (3%–5.1%) [8, 9], iatrogenic scleral break (2%) [10], accidental subretinal fluid drainage (5%–8%) [8, 10], retinal breaks (0.54%–4%) [8], choroidal hemorrhage (2%) [10], retinal incarceration (2.2%–3%) [8], explant exposure (6%) [10], macular pucker (2%) [6, 9], and PVR (5%–21%) [6, 9].

Moreover, breaks between the equator and major vessel arcades are commonly supported by one or more large radial sponges that need a surgical familiarity for the correct sponge placement with a potential risk of compression of the vortex veins [1].

In a previous study, Uemura and Nakao showed that, in the eyes with uncomplicated RRD caused by a posterior retinal break, both procedures SB and PPV had a similar visual recovery but the vitrectomized eyes had less severe intraoperative complications compared with SB [16].

Vitrectomy offers some advantages such as easy access to intermediate retinal breaks and greater familiarity with this technique compared with SB [1, 4]. However, PPV is known to cause cataract progression and may cause several other complications such as glaucoma (8.9%) [17], choroidal hemorrhage (0.8%) [18], diplopia/EOM dysfunction (0.5%–7%) [6, 9], cataract (70%) [9], macular pucker (9%) [7], postoperative PVR (6%–18%) [6, 7], and iatrogenic retinal breaks (6%–15.7%) [7, 19].

Our small gauge-modified vitrectomy showed a single-operation anatomical success rate of 94%, which is consistent with the rates of 74–93.9% for repair of primary RRD in other reports of conventional small-gauge vitrectomy [20–22] and did not cause significant progression of cataract.

We observed only one case with intraoperative hemorrhage requiring conversion to standard PPV and that was excluded by the analysis.

Moreover, no statistically significant difference between eyes with superior retinal break(s) and eyes with inferior retinal break(s) in terms of primary anatomical success rate (*P*=0.534) was noted in our study. However, this topic is still controversial; in fact, despite Goto et al. [23] reported that inferior retinal breaks were significantly associated with a lower success anatomic comparing with superior retinal breaks (80% versus 98%, *P*=0.012), the other authors [24] showed inferior breaks do not represent a risk factor for worse anatomical and functional results (96.5% versus 93.3%, respectively, in superior and inferior retinal breaks).

It is well established that vitrectomy increases lens opacity in most eyes when assessed at 6 months regardless of the caliber of the instrument [25].

In our study, no progression of nuclear sclerosis was observed through the 12 months of follow-up, and the NDUs at 12 months did not differ between the vitrectomized and fellow eyes. Our result is consistent with previous studies that found no progression of cataract in the eyes that had undergone removal of the ERM without vitrectomy [26, 27].

The mechanism underlying cataract progression after vitrectomy is not completely understood. One hypothesis is that, in the absence of vitreous gel, molecular oxygen from the retinal vasculature reaches the lens and promotes oxidative damage of the lens nucleus and nuclear sclerotic cataract [28]. According to this hypothesis, the very limited amount of vitreous removal in our modified vitrectomy, with core and vitreous base preservation, could explain the absence of progression of cataract.

Our choice to use air as tamponade was supported by findings of previous studies reporting favorable results with air tamponade in the management of RRD, with a single-operative success rate from 84.38% to 94.4% [29]. Air used as gas tamponade showed no inferior results to long-acting gas because of the adhesion between retina and retinal pigment epithelium (RPE) occurring within 24 hours. Moreover, long-acting and expansive gas could cause vitreous disturbance and increase the risks of elevated IOP, PVR, and new or missed tears [30].

Despite the recent evolution of vitreoretinal surgical techniques, the incidence of “new” retinal breaks has been reported for small-gauge PPV in up to 15.7% of eyes [19]. Although the sample number was small in our study, we found new retinal breaks in only 1 eye (3%) during the 12-month follow-up and suggests that the residual vitreous does not cause secondary vitreous traction in these eyes. Similarly, previous reports of no vitrectomizing vitreous surgery for ERM have also reported no new retinal breaks during the long follow-up [31].

In our series, ERM developed in 3% of eyes. This is a lower percentage than the 3.6–12.8% reported by others [32, 33]. The most likely explanation for the development of ERM is that the retinal pigment epithelial cells migrate to the surface of the posterior pole of the retina by diffusing into the vitreous cavity through the break or through fibrosis [33]. In our modified technique, the remaining vitreous probably prevented such diffusion into the vitreous cavity.

Furthermore, a potential advantage of our new technique of “localized vitrectomy,” without core and vitreous base removing, is that it still allows intravitreal injections in the eyes experiencing the onset of neovascular age-related macular degeneration, which can be difficult to treat after previous conventional PPV. Experimental studies have shown a reduction in the intravitreal half-life of drugs in the vitrectomized eyes due to significantly faster clearance rates of the drugs after vitrectomy, which could make them less effective [34, 35], and it may require more frequent treatment regimen of anti-VEGF therapy [34, 35].

The main limitations of this study are the small number of patients enrolled and the lack of an interventional control group. Large studies should also evaluate the efficacy and rate of postoperative complications.

## 5. Conclusion

“Localized vitrectomy” seems to be an effective surgical procedure to treat uncomplicated macula-on primary RRD with intermediate break(s), marked vitreous traction, and PVD in the phakic eyes, achieving a high anatomical success rates without progression of cataract.

## Figures and Tables

**Table 1:** NDUs at the baseline and at 3, 6, and 12 months after surgery.

NDUs (range, 0 to 255 steps) (mean ± SD)	Vitrectomy group	Control group	*P* ^*∗*^
Baseline	68 ± 12	69 ± 14	0.933
3 months	70 ± 12	69 ± 16	0.779
6 months	72 ± 18	71 ± 11	0.875
12 months	73 ± 15	72 ± 9	0.724

^*∗*^ *t*-test.

## Data Availability

The data used to support the findings of this study are included within the article.

## References

[1] Kreissig I. (2003). View 1: minimal segmental buckling without drainage. *British Journal of Ophthalmology*.

[2] McLeod D. (2004). Is it time to call time on the scleral buckle?. *British Journal of Ophthalmology*.

[3] Kellner L., Wimpissinger B., Stolba U., Brannath W., Binder S. (2007). 25-gauge vs 20-gauge system for pars plana vitrectomy: a prospective randomised clinical trial. *British Journal of Ophthalmology*.

[4] Feltgen N., Weiss C., Wolf S., Ottenberg D., Heimann H., SPR Study Group (2007). Scleral buckling versus primary vitrectomy in rhegmatogenous retinal detachment study (SPR Study): recruitment list evaluation. Study report no. 2. *Graefe’s Archive for Clinical and Experimental Ophthalmology*.

[5] Heimann H., Baertz-Schmidt K. U., Bronfeld N., Weiss C., Hilgers R. D., Foerster M. H. (2007). Scleral buckling versus primary vitrectomy in rhegmatogenous retinal detachment: a prospective randomized multicenter clinical study. *Ophthalmology*.

[6] Steel D. (2014). Retinal detachment. *BMJ Clinical Evidence*.

[7] The SPR Study Study Group (2003). View 2: the case for primary vitrectomy. *British Journal of Ophthalmology*.

[8] Holz E. R., Mieler W. F. (2003). View 3: the case for pneumatic retinopexy. *British Journal of Ophthalmology*.

[9] Lv Z., Li Y., Wu Y., Qu Y. (2015). Surgical complications of primary rhegmatogenous retinal detachment: a meta-analysis. *PLoS One*.

[10] Abdullah A. S., Jan S., Qureshi M. S., Khan M. T., Khan M. D. (2010). Complications of conventional scleral buckling occurring during and after treatment of rhegmatogenous retinal detachment. *Journal of the College of Physicians and Surgeons Pakistan*.

[11] Seider M. I., Naseri A., Stewart J. M. (2013). Cost comparison of scleral buckle versus vitrectomy for rhegmatogenous retinal detachment repair. *American Journal of Ophthalmology*.

[12] Thompson J. T., Glaser B. M., Sjaarda R. N., Murphy R. P. (1995). Progression of nuclear sclerosis and long-term visual results of vitrectomy with transforming growth factor beta-2 for macular holes. *American Journal of Ophthalmology*.

[13] Sawa M., Saito Y., Hayashi A., Kusaka S., Ohji M., Tano Y. (2001). Assessment of nuclear sclerosis after nonvitrectomizing vitreous surgery. *American Journal of Ophthalmology*.

[14] Vivino M. A., Chintalagiri S., Trus B., Datiles M. (1993). Development of a Scheimpflug slit lamp camera system for quantitative densitometric analysis. *Eye*.

[15] Chan C. K., Lin S. G., Nuthi A. S., Salib D. M. (2008). Pneumatic retinopexy for the repair of retinal detachments: a comprehensive review (1986–2007). *Survey of Ophthalmology*.

[16] Uemura A., Nakao K. (1995). A comparison between scleral buckling procedure and vitrectomy for the management of uncomplicated retinal detachment caused by posterior retinal break. *Nippon Ganka Gakkai Zasshi*.

[17] Mansukhani S. A., Barkmeier A. J., Bakri S. J. (2018). The risk of primary open angle glaucoma following vitreoretinal surgery: a population-based study. *American Journal of Ophthalmology*.

[18] Reibaldi M., Longo A., Romano M. R. (2015). Delayed suprachoroidal hemorrhage after pars plana vitrectomy: five-year results of a retrospective multicenter cohort study. *American Journal of Ophthalmology*.

[19] Ehrlich R., Goh Y. W., Ahmad N., Polkinghorne P. (2012). Retinal breaks in small-gauge pars plana vitrectomy. *American Journal of Ophthalmology*.

[20] Lewis S. A., Miller D. M., Riemann C. D., Foster R. E., Petersen M. R. (2011). Comparison of 20-, 23-, and 25-gauge pars plana vitrectomy in pseudophakic rhegmatogenous retinal detachment repair. *Ophthalmic Surgery, Lasers, and Imaging*.

[21] Rezar S., Sacu S., Blum R., Eibenberger K., Schmidt-Erfurth U., Georgopoulos M. (2016). Macula-on versus macula-off pseudophakicrhegmatogenous retinal detachment following primary 23-gaugevitrectomy plus endotamponade. *Current Eye Research*.

[22] Mehta S., Blinder K. J., Shah G. K., Grand M. G. (2011). Pars plana vitrectomy versus combined pars plana vitrectomy and scleral buckle for primary repair of rhegmatogenous retinal detachment. *Canadian Journal of Ophthalmology*.

[23] Goto T., Nakagomi T., Iijima H. (2013). A comparison of the anatomic successes of primary vitrectomy for rhegmatogenous retinal detachment with superior and inferior breaks. *Acta Ophthalmologica*.

[24] Stavrakas P., Tranos P., Androu A. (2017). Anatomical and functional results following 23-Gauge primary pars plana vitrectomy for rhegmatougenous retinal detachment: superior versus inferior breaks. *Journal of Ophthalmology*.

[25] Feng H., Adelman R. A. (2014). Cataract formation following vitreoretinal procedures. *Clinical Ophthalmology*.

[26] Reibaldi M., Longo A., Avitabile T. (2015). Transconjunctival non vitrectomizing vitreous surgery versus 25-gauge vitrectomy in patients with epiretinal membrane: a prospective randomized study. *Retina*.

[27] Sawa M., Ohji M., Kusaka S. (2005). Nonvitrectomizing vitreous surgery for epiretinal membrane long-term follow-up. *Ophthalmology*.

[28] Milazzo S. (2014). Pathogenesis of cataract after vitrectomy. *French Journal of Ophthalmology*.

[29] Zhou C., Qiu Q., Zheng Z. (2015). Air versus gas tamponade in rhegmatogenous retinal detachment with inferior breaks after 23-gauge pars plana vitrectomy: a prospective, randomized comparative interventional study. *Retina*.

[30] Pak K. Y., Lee S. J., Kwon H. J., Park S. W., Byon I. S., Lee J. E. (2017). Use of air as gas tamponade in rhegmatogenous retinal detachment. *Journal of Ophthalmology*.

[31] Saito Y., Lewis J. M., Park I. (1999). Nonvitrectomizing vitreous surgery: a strategy to prevent postoperative nuclear sclerosis. *Ophthalmology*.

[32] Katira R. C., Zamani M., Berinstein D. M., Garfinkel R. A. (2008). Incidence and characteristics of macular pucker formation after primary retinal detachment repair by pars plana vitrectomy alone. *Retina*.

[33] Nam K. Y., Kim J. Y. (2015). Effect of internal limiting membrane peeling on the development of epiretinal membrane after pars plana vitrectomy for primary rhegmatogenous retinal detachment. *Retina*.

[34] Gisladottir S., Loftsson T., Stefansson E. (2009). Diffusion characteristics of vitreous humor and saline solution follow the Stokes Einstein equation. *Graefe’s Archive for Clinical and Experimental Ophthalmology*.

[35] Christoforidis J. B., Williams M. M., Wang J. (2013). Anatomic and pharmacokinetic properties of intravitreal bevacizumab after vitrectomy and lensectomy. *Retina*.

